# Multimodal and Multidimensional Tactile Perception for Universal Robotic Sophisticated Tasks

**DOI:** 10.34133/research.1477

**Published:** 2026-11-06

**Authors:** Qian Mao, Yichen Qin, Zijian Liao, Rong Zhu

**Affiliations:** State Key Laboratory of Precision Measurement Technology and Instruments, Department of Precision Instrument, Tsinghua University, Beijing, China.

## Abstract

Human hands have rich tactile senses with multimodal and multidimensional perceptions for hand dexterous manipulation. However, current robotic tactile sensory modality and dimensionality are deficient and hardly meet the harsh demands for diverse manipulation tasks. Here, we report a multimodal and multidimensional tactile sensor based on unified thermo-sensation, enabling comprehensive perceptions of contact, matter type, slip/slide, texture/scratch, temperature, and multidimensional force to meet the general-purpose perceptual requirement across diverse sophisticated robotic tasks. The sensor is compact, flexible, ultralight (0.33 g), and integrated on the robot’s fingertip. Ultrafast contact and slip detections (~5 ms) of the sensor enable instantaneous action triggering of robots to ensure agile manipulations, while its precise texture/scratch detection (at the ~20 μm scale) surpasses human touch sensitivity. The sensor also provides decoupled and high-precision measurements of 3-axis forces with fast response, low hysteresis, and low crosstalk, ensuring dexterous action control of robots. Furthermore, we propose a framework of tactile perception–control synergy, endowing robots with self-adaptive action-on-feeling like humans. Leveraging the multimodal and multidimensional tactile perception as well as the synergy with adaptive control, robots autonomously accomplish various sophisticated tasks of precise scratch inspection, fine assembly, and plug-socket insertion, etc.

## Introduction

Intelligent robots are increasingly entering our daily lives and industrial settings to support household services and industrial automation, where they need to execute an increasingly wide array of complex tasks. Although visual perception has been widely employed in robotic systems for object localization and recognition [1–3], its performance in complex environments can be limited by lighting variations and occlusions [2–4]. Furthermore, for intricate manipulation tasks such as a precise assembly of components or quality inspection of workpieces, it is often necessary to perceive object contact states, surface textures, and contact forces during manipulation. Consequently, tactile perception has emerged as a crucial means of complementing visual perception. Tactile sensors provide robots with real-time tactile feedback. Moreover, tactile sensors enable robots to perceive objects’ physical properties [5,6], slippage [7,8], and textures/scratches [9,10], thereby enhancing the robotic understanding of both its environment and objects in touch. Such tactile perception plays a pivotal role in executing intricate manipulation tasks.

In recent years, tactile sensors based on piezoresistive [11–15], capacitive [16–18], piezoelectric [18–21], triboelectric [22–25], magnetic [26–28], and vision-based [29–32] principles have been developed for robotic tactile sensing in robotic grasping, object sorting, and human–robot interaction. However, most tactile sensors primarily focus on limited perceptual dimension, such as force or temperature sensing, which hinders robots from comprehensively surveying the interactions in complex tasks. Though multimodal and multidimensional tactile sensors have been investigated via integrating distinct sensing principles to perceive force, contact/slip, temperature, material properties, and surface features [5,6,22,33–36], the multimodal/multidimensional integration of tactile sensors is still low and challenging.

Dexterous manipulation imposes sophisticated perception and control: Robots need to rapidly and precisely perceive interface states while simultaneously regulating multidimensional contact forces throughout closed-loop operations. Existing perceptual modality and dimensionality of current tactile sensors are deficient. Limited by existing sensing principle, the single-principle provides restricted tactile modality [6,22], while gathering distinct sensing mechanisms into single sensor for multimodality raises its complexity and thus degrades its integration [5,35,36]. In order to meet the general-purpose perceptual requirement of intelligent robots across sophisticated tasks, it is necessary to develop a compact and highly integrated tactile sensor integrating multimodal and multidimensional perceptions, and build an adaptive perception–control synergy framework, which remains a major challenge.

To address the challenge, we develop a compact and highly integrated tactile sensor (fingertip size, 0.33 g) based on unified thermo-sensation, gathering multimodal and multidimensional perceptions of contact, matter type, slip/slide, texture/scratch, temperature, and 3-axis force, aiming at diverse robotic manipulation tasks (shown in Fig. 1). During a common manipulation process, the robotic hand experiences the temporal senses of uncontact, contact, grasp, and other dexterous manipulations, such as rotation, insertion, material identification, and surface inspection. In this process, the tactile sensor first needs to swiftly sense the contact state and recognize object; then it needs to quickly sense the slip/slide state or delicately inspect the surface of the object (texture/scratch) in hand. Furthermore, simultaneous and independent measurement of 3-axis force is also required to complete dexterous action control. Our sensor integrates multimodal tactile sensing and 3-axis force measurement by using unified thermo-sensation principle and a compact sensory architecture to meet these diverse manipulation requirements. It is structured with a multimodal interface sensing layer and a multidimensional force sensing layer. The surface sensing is based on the principle of interfacial heat transfer by capturing the intensity change of heat transfer from the sensor to the environment/object in touch, achieving ultrasensitive and ultrafast interface sensing to contact state, slip/slide, texture/scratch, etc. Three-axis force measurement is based on scattered thermo-sensation to capture force-induced spatial strain in a flexible piezo-thermic material, achieving decoupled measurements of normal and shear forces. Benefiting from unified thermo-sensation, our multimodal and multidimensional tactile sensor is compact, highly integrated, and with high precision, fast response, low hysteresis, and low crosstalk. We further build a framework of tactile perception–control synergy to mimic humans’ nature of delicate “action-on-feeling”, enabling robots to adaptively accomplish diverse challenging tasks in the real world, such as automated surface defect/scratch inspection on uneven workpieces, automated screw assembly, and automated plug–socket insertion (Movie S1).

**Fig. 1.**
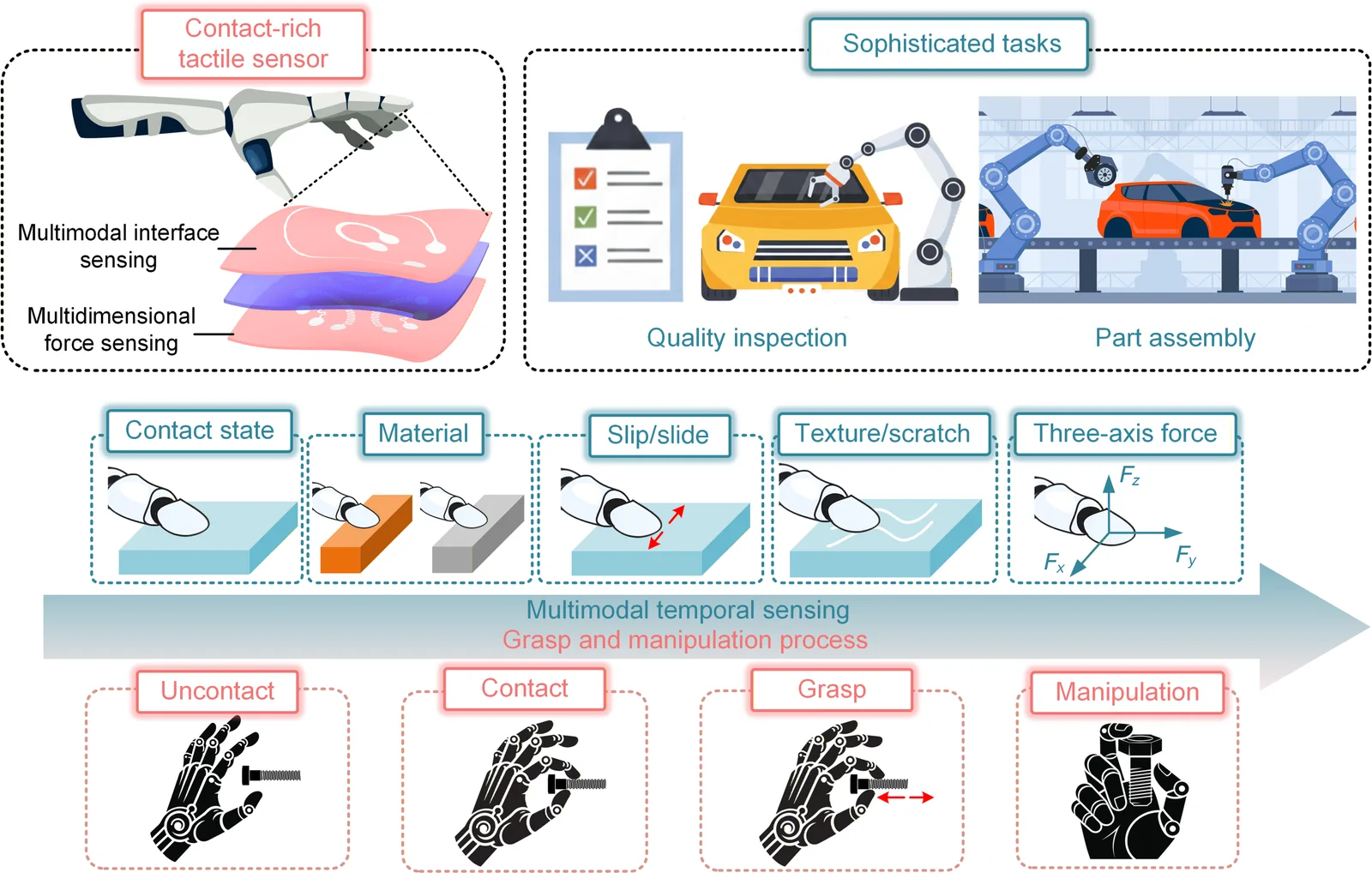
Sophisticated tasks enabled by multimodal and multidimensional tactile sensing.

## Results and Discussion

### Working principle of multimodal and multidimensional tactile sensor

The proposed tactile sensor comprises an interface sensing layer (on the top), a porous polydimethylsiloxane (PDMS) with silver (in the middle), and a 3-axis force sensing layer (at the bottom) (shown in Fig. 2A). The interface sensing layer consists of thin-film thermistors composed of Cr (30 nm) and Pt (120 nm) fabricated on a flexible polyimide, comprising concentric circles with radii of 3 mm (denoted as cold film) and 1 mm (denoted as hot film). The bottom 3-axis force sensing layer contains 3 scattered sensing units, each comprising a cold film with a radius of 2 mm and a hot film with a radius of 0.8 mm (details in Fig. S1). Figure 2B illustrates the principle of contact, material, slip/slide, and texture/scratch sensing of the interface sensing layer. The hot film is electrically heated (about 15 °C higher than ambient temperature) and transfers the heat with the surroundings. When the sensor contacts an object, the heat exchange between the hot film and the object intensifies. When a slide/slip occurs, the contact area between the sensor and the object shifts from a heated region to an unheated one, resulting in a change in the intensity of heat exchange, i.e., a variation detected by the hot film. When the object in contact with the interface sensing layer changes from a scratch-free area to a scratched area, the micro scratch disturbs the heat-transfer field from the sensor to the object, which is detected by the hot film, thus allowing the scratch detection on the object’s surface. The monolithically integrated cold film is unheated, serving as an ambient/object temperature sensor, and also makes a temperature compensation for the hot film. Figure 2C demonstrates the interface voltage response (denoted as *U_i_*) to object contact (the sensor’s conditioning circuit is shown in Fig. S2), with a fast response time of 6 ms. Figure 2D exhibits the interface voltage response (*U_i_*) when the interface sensing layer contacts objects with different materials. Table S1 lists the thermal conductivities of these materials. Object materials with different thermal conductivities induce different interfacial heat-transfer responses; consequently, the hot film on the interface sensing layer enables the identification of the material type. For the object materials with similar thermal properties, the surface texture and mechanical properties can provide supplementary tactile cues in material identification. The sensor is also capable of rapidly detecting sliding/slip, with a response time of 5 ms (Fig. 2E). Figure 2F demonstrates the signal when the sensor slides across the surface of an object (stainless steel) at a speed of 1 mm/s, and this signal can subsequently be utilized to detect surface texture or scratches of the object. A fast Fourier transform performed on this signal yields the frequency spectrum shown in Fig. 2G, wherein the peak frequency of 4 Hz indicates that the surface texture width of the object is (1 mm/s) / 4 Hz = 250 μm. The surface scratches can also be identified (dashed boxes in Fig. 2F) by using a moving window detection method, which will be described in detail later. We adopt a strategy of time-division and frequency-division detection to share the interface sensing signal for multimodal tactile perceptions in manipulation processes. Material identification utilizes the static interface signal under a firm grip condition, whereas the contact and slip events are detected from the transient changes of the interface signal. The scratches are detected from the localized fluctuations during the controlled sliding processes, meanwhile the periodic texture provides a frequency feature in the interface signal that can be distinguished from the scratch-induced abrupt signals.

**Fig. 2.**
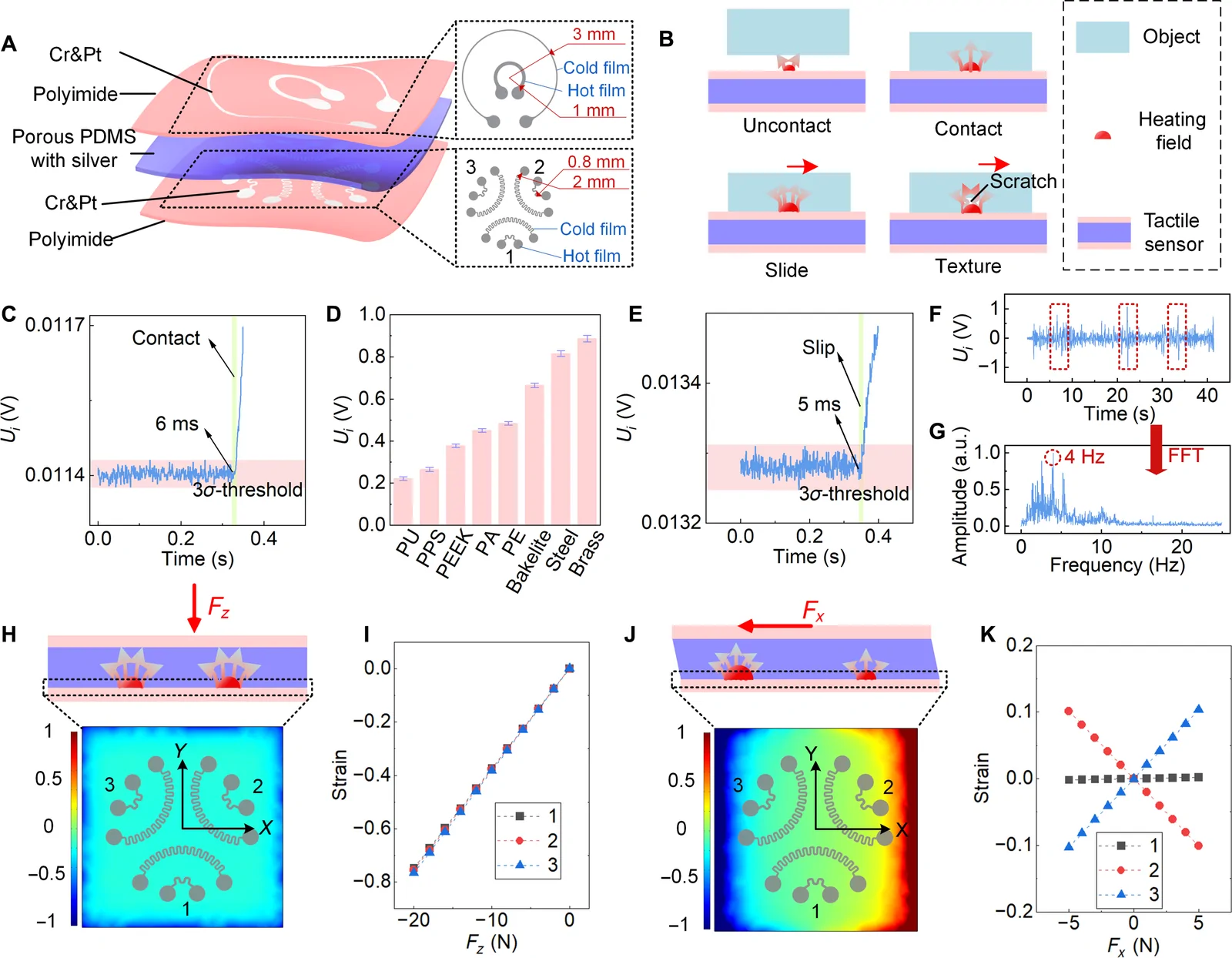
The working principle of the multimodal and multidimensional tactile sensor. (A) The structure of the tactile sensor, which consists of an interface sensing layer on the top, a porous material in the middle, and a 3-axis force sensing layer at the bottom. The interface sensing layer contains a sensing unit, and the 3-axis force sensing layer contains 3 sensing units. PDMS, polydimethylsiloxane. (B) Working principle of the interface sensing layer. (C) The contact-state sensing of the interface sensing layer. (D) The material sensing of the interface sensing layer. PU, polyurethane; PPS, polyphenylene sulfide; PEEK, polyether ether ketone; PA, polyamide; PE, polyethylene. (E) The sliding/slip sensing of the interface sensing layer. (F) The scratch sensing of the interface sensing layer. (G) The texture sensing of the interface sensing layer. (H) Schematic diagram of the sensor deformation and the strain field distribution under normal force (*F_z_*). (I) Strains on 3 sensing units under different normal forces (*F_z_*). (J) Schematic diagram of the sensor deformation and the strain field distribution under shear force (*F_x_*). (K) Strains on 3 sensing units under different shear forces (*F_x_*).

The 3-axis force sensing layer measures 3-axis force by capturing the spatial strain of the porous material (i.e., piezo-thermic material) utilizing 3 scattered hot films at the bottom sensing layer. The force causes a compression of the porous material, reducing the air content and thus affecting its thermal conductivity and heat-transfer intensity in local areas, which are detected by 3 scattered hot films underneath the porous material. Figure 2H illustrates the deformation of the porous material under normal pressure (*F_z_*) and the force-induced spatial strain that alters the heat transfer of the hot films. As shown in Fig. 2I, under normal pressure (*F_z_*), the local strains at the 3 sensing units are the same and exhibit the same trend with changes in *F_z_*. Figure 2J illustrates the deformation of the porous material under shear force (*F_x_*) and the force-induced spatial strain that alters the heat transfer of the hot films. The shear force causes the differences in the strains at the locations of the 3 sensing units. Figure 2K exhibits the strain variations under different shear forces (*F_x_*). Sensing units 2 and 3 exhibit opposite trends in strain because they are symmetric about the *y*-axis. Sensing unit 1, located along the *y*-axis, shows almost no change. Therefore, different forces along 3 axes cause different spatial strain fields in the porous material, which can be detected by the 3 scattered sensing units.

Based on the above sensor design, we fabricate the patterned thin-film thermistors (Pt/Cr) on a flexible printed circuit board (polyimide) using photolithography and sputtering to obtain the interface and force sensing layers. The porous PDMS with silver is fabricated using a sacrificial template method and sandwiched between the interface and force sensing layers. The detailed fabrication process is described in the “Materials and Methods” section and Fig. S3. The fabricated sensor is shown in Fig. S4.

### Three-axis force measurement of the tactile sensor

As mentioned above, the tactile sensor is capable of 3-axis force measurements simultaneously and independently. Figure 3A illustrates the coordinate system of the sensor and the layout of the 3 sensing units at the bottom sensing layer. Figure 3B exhibits the voltage responses (denoted as *U*_*p*1_, *U*_*p*2_, and *U*_*p*3_) of the 3-axis force sensing layer under normal force (*F_z_*) during loading and unloading, respectively. The results indicate a good consistency among the 3 sensing units, exhibiting a low hysteresis error (3.3%). The response time and recovery time of the pressure sensor are 44 ms and 52 ms, respectively. The minimum detection limit is 0.003 N for normal force *F_z_* (shown in Fig. 3C), and the minimum detection limits for tangential forces *F_x_* and *F_y_* are 0.017 N and 0.018 N, respectively (shown in Fig. S5). Furthermore, we test the sensor’s repeatability in 5,000 cycles of −20 N (Fig. 3D), where both the interface sensor and the 3-axis force sensing layer exhibit good stability and reliability. The pressure sensor is also tested under 3-axis forces in real time, with an experiment setup shown in Fig. S6. Figure 3E presents the voltage responses of the 3 sensing units under 3-dimensional forces. When *F_z_* of −4 N is applied, the 3 sensing units (*U*_*p*1_, *U*_*p*2_, and *U*_*p*3_) change synchronously. When *F_x_* of −3 N and *F_z_* of −4 N are applied, the voltage *U*_*p*2_ of unit 2 decreases while the voltage *U*_*p*3_ of unit 3 increases, indicating that the shear force is along the negative *x*-axis. Since the sensing unit 1 is located on the *y*-axis, *U*_*p*1_ remains essentially unchanged. When *F_x_* of 3 N and *F_z_* of −4 N are applied, the opposite signal change is observed. When *F_y_* of −3 N and *F_z_* of −4 N are applied, the voltage *U*_*p*1_ of unit 1 increases while the voltages *U*_*p*2_ and *U*_*p*3_ of units 2 and 3 decrease, indicating that the shear force is along the negative *y*-axis. When *F_y_* of 3 N and *F_z_* of −4 N are applied, the opposite signal change is observed. In order to figure out the 3-axis force from the voltages *U*_*p*1_, *U*_*p*2_, and *U*_*p*3_ of the 3-axis force sensing layer, we employ a fully connected neural network as the calibration model (detailed in the “Materials and Methods” section and Supplementary Notes). Figures 3F to H present the results of the sensor’s 3-axis force measurement. The sensor’s ranges of the normal force *F_z_*, shear force *F_x_*, and *F_y_* are −20~0 N, −5~5 N, and −5~5 N, respectively. The correlation coefficients (*R*^2^) between the true and measured values are 0.999, 0.993, and 0.994 for *F_z_*, *F_x_*, and *F_y_*, respectively, which indicates a high consistency between the measured 3-axis forces and the true forces. Furthermore, the error results shown in the inset demonstrate a high accuracy in the 3-axis force measurement, with root-mean-square errors of 0.16 N, 0.17 N, and 0.18 N for *F_z_*, *F_x_*, and *F_y_*, respectively. Additionally, the coupling errors between different axes are low (<2.3%) (Fig. S7), indicating that the sensor is capable of simultaneous and independent measurements on 3-axis forces.

**Fig. 3.**
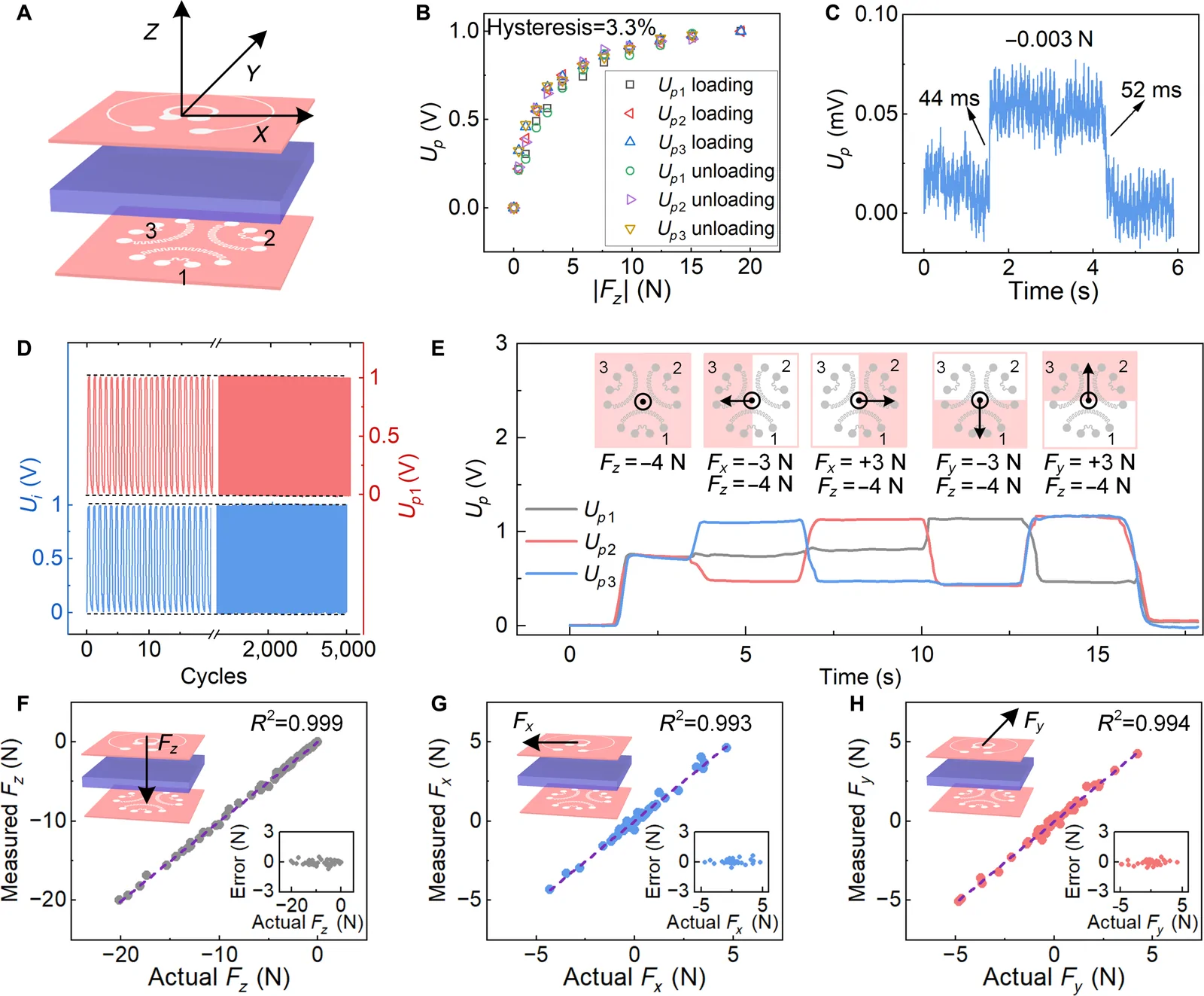
Three-axis force measurement of the tactile sensor. (A) The coordinate system of the sensor and the layout of the three 3-axis force sensing units. (B) The voltage responses of the 3 sensing units under normal force (*F_z_*) loading and unloading. (C) Response time, recovery time, and detection limit of the 3-axis force sensing. (D) Repeatability of the sensor with −20 N (*F_z_*) under 5,000 cycles. (E) The voltage responses of the three 3-axis force sensing units versus 3-axis forces. (F) Performance of the 3-axis force sensing for *F_z_*. (G) Performance of the 3-axis force sensing for *F_x_*. (H) Performance of the 3-axis force sensing for *F_y_*.

We also test the effect of temperature influence on the interface sensing layer and the 3-axis force sensing layer, showing that the tactile sensor is almost immune to ambient temperature variation (Fig. S8). The long-term drift of the tactile sensor is evaluated and exhibits low drift errors of 1.8% and 1.1% in force-sensing and interface-sensing outputs, respectively (Fig. S9). Furthermore, the coupling crosstalk between the interface sensing layer and the 3-axis force sensing layer is also tested and indicates a low crosstalk (<4.0%) (Figs. S10 and S11). The above results demonstrate that the multimodal and multidimensional tactile sensor is competent to detect contact, matter, texture/scratch, temperature, and 3-axis forces independently and has a low detection limit, fast response speed, high reliability, and good stability. It is noted that the ultrafast detections of contact and slip (~5 ms) ensure the instantaneous action trigger of robots. The precise and fast measurements of 3-axis forces ensure multidimensional dexterous action control for robotic manipulation.

### Surface scratch detection by the tactile sensor

In the industrial task of workpiece quality inspection, the detection of minor defects such as surface scratches is among the most challenging issues to automate. Existing automated inspection solutions mainly rely on vision [37–40]. However, in real-world production environments, visual methods are easily affected by variation in lighting, viewing angle limitations, surface reflectivity, and material differences. For workpieces with highly reflective metals, complex textures, or non-flat curved surfaces, minute scratches are difficult to identify reliably [41,42]. Under conventional industrial configurations, the detection of scratches is hard to automate [43,44]. Currently, the final determination of surface defects on production lines (e.g., auto production) still relies on manual touch and experience, which limits the automation level of quality inspection.

Our proposed tactile sensor can implement precise scratch detection, even on uneven surfaces. Figure 4A illustrates how to extract the scratch signals from the interface signal *U_i_* when the sensor slides on the object surface at a speed of 1 mm/s. First, the interface signal is filtered to remove the baseline (the baseline can be utilized to identify contact, matter, and slip/slide). Then, a moving window detection method is used to calculate the standard deviation (*σ*_w_) of the signal within the window length and the overall standard deviation of the signal (*σ*_a_). If *σ*_w_ >3*σ*_a_, the signal within the window length is recognized as a scratch signal; otherwise, it is a non-scratch signal. A detailed description of the method is provided in the “Materials and Methods” section and Figs. S15 and S16. To verify the scratch detection method, we select 4 typical materials, aluminum alloy, stainless steel, polytetrafluoroethylene (teflon), and resin, on which the scratches with varying widths are detected (photographs of the scratches are shown in Fig. S12). According to their widths, the scratches are categorized as small scratches (20 to 40 μm, beyond the limit of human touch sensitivity), medium scratches (40 to 70 μm), and large scratches (>70 μm). We test 30 scratches on the surfaces of the 4 materials. Each scratch-width category (small, medium, and large) contains 10 scratches, and each scratch is tested 3 times, yielding 90 testing samples in total. Figure 4B demonstrates the scratch detection results of the true-positive rate (refers to the scratch being accurately identified, abbreviated as TP), false-positive rate (non-scratch signal is misidentified as scratch signal, abbreviated as FP), and false-negative rate (scratch signal is misidentified as non-scratch signal, abbreviated as FN). For small, medium, and large scratches, the true-positive rates are 90.0%, 93.3%, and 100.0%, respectively. The detection accuracy increases with the scratch width. Figures 4C to F display the photographs of small scratches on 4 materials and the corresponding scratch signals, respectively. For these 4 materials (aluminum alloy, stainless steel, teflon, and resin), the tactile sensor detects the scratches with widths down to 27.4, 33.6, 24.2, and 26.2 μm, respectively. It is noted that the scratches used in the experiments are created manually, whose dimensions cannot be precisely controlled across materials. As a result, the detected scratch widths do not refer to material-specific detection limit. In our experiments, the sensor retains effective scratch detections after repeated contact and sliding on scratched surfaces. We compare the interface signals before and after repetitive scratch detections (90 trials in total). The interface signals retain the scratch detection capability as shown in Fig. S13. In the condition of larger scratches with protruding burrs, the scratch detection needs to combine vision- and touch-based detections. Vision is utilized to screen the large scratches via noncontact manner, while the small scratches hardly resolved by vision are detected by the touch-based detection. This vision–touch fusion can enhance the scratch detection rate and reduce the risk of sensor damage during contact inspection.

**Fig. 4.**
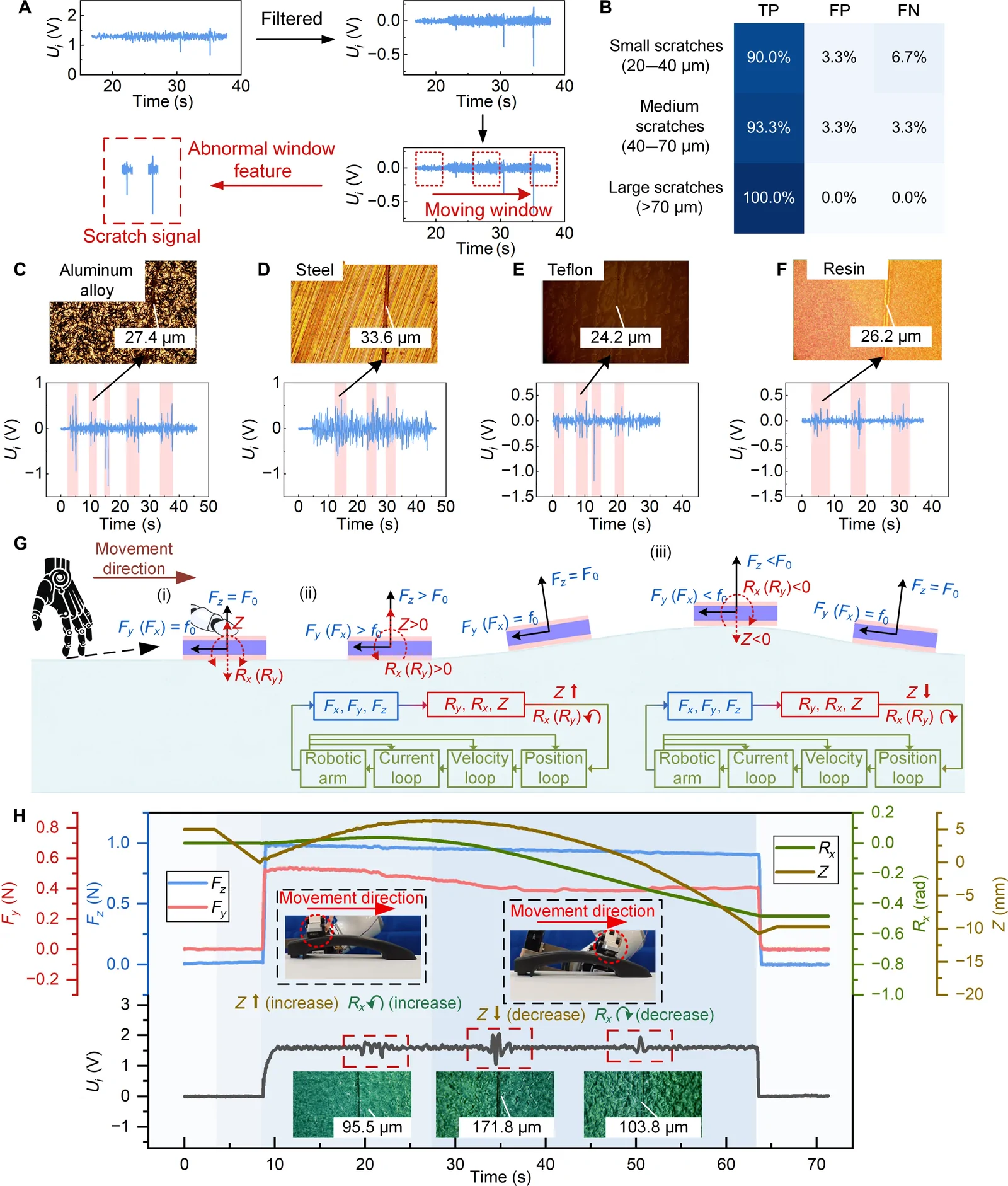
Scratch-inspection task by robotic tactile perception–control synergy. (A) The schematic diagram of the scratch signal detection method. (B) The results of the true-positive rate (TP), false-positive rate (FP), and false-negative rate (FN) of small scratches (20 to 40 μm), medium scratches (40 to 70 μm), and large scratches (>70 μm), respectively. (C to F) Micrographs and detection signals of small scratches on aluminum alloy, steel, teflon, and resin, respectively. (G) The schematic diagram and control flowchart of the robotic finger with the tactile sensor sliding on the workpiece surface. (H) Responses of the tactile sensor and the robotic arm during scratch detection on an uneven car door handle.

### Robotic tactile perception–control synergy for scratch/defect inspection

As mentioned above, workpieces on industry production lines require surface inspection to detect scratches or other defects. To automate surface inspection, tactile perception and control are both essential. Tactile perception provides a precise detection of scratches; meanwhile, 3-axis force control ensures adaptive alignment of the robotic fingertip to make good contact with the workpiece and parallelly slide along its surface. This tactile perception–control synergy is crucial to ensure the safety and success of precise scratch detection. We equip the tactile sensor on a robotic hand to perform scratch detection on a workpiece. Figure 4G illustrates a schematic diagram and control flowchart of the robotic hand with the sensor sliding on the workpiece surface. The schematic diagram of the robotic control system for the workpiece defect inspection is illustrated in Fig. S14. Since workpiece surfaces are often uneven and have undulations, 3-axis force control is needed to achieve adaptive good contact between the tactile sensor and the workpiece. From Fig. 4G, the tactile sensor perceives the shear force *F_y_* or *F_x_* and normal force *F_z_* in real time. In an ideal circumstance, the robotic finger slides parallelly on the workpiece surface with an appropriate force *F_z_* and a sliding friction force *F_y_* or *F_x_*. However, uneven surface or misaligned surface in real scenarios alters the shear and normal forces, which can be detected by the tactile sensor. Accordingly, the robot arm is maneuvered to move in the *z*-direction (denoted as *Z*) and rotate in the *x*-axis (*R_x_*) or the *y*-axis (*R_y_*) to align the contact. Therefore, even on an uneven workpiece surface, the robot can adaptively adjust its posture and autonomously accomplish the scratch-inspection task. To ensure safe and dexterous contact between the sensor and workpiece, we hypothesize that the interaction between the tactile sensor and the workpiece follows a virtual spring-damping kinetic mechanism and propose the following interactive control law:

$$Z=k_{1}\left(F_{z}-F_{0}\right)+\mu_{1}\overset{\cdot }{Z}$$ (1)

$$R_{x}=k_{2}\left(F_{y}-f_{0}\right)+\mu_{2}\overset{\cdot }{R_{x}}$$ (2)

$$R_{y}=k_{3}\left(F_{x}-f_{0}\right)+\mu_{3}\overset{\cdot }{R_{y}}$$ (3)

where *k*_1_, *k*_2_, *k*_3_, *μ*_1_, *μ*_2_, and *μ*_3_ are coefficients related to the robot arm control (detailed in Supplementary Notes). $\overset{\cdot }{Z}$, $\overset{\cdot }{R_{x}}$, and $\overset{\cdot }{R_{y}}$ are the velocity in the *Z* direction and the rotational angular velocities in the *X* and *Y* directions, respectively. *F*_0_ and *f*_0_ are the support force and the sliding friction force when the sensor and the object are in good contact, respectively (as shown in state (i) in Fig. 4G). *F*_0_ is determined by trial to provide an appropriate pressing force between the sensor and the workpiece, ensuring good scratch detection without damaging either the workpiece or the sensor. *f*_0_ is autonomously determined by the robot in its initial self-alignment. During the initial self-alignment process, the robotic finger with the sensor reciprocates on the workpiece surface. The robotic arm adaptively adjusts its translation and rotation to make the shear forces from the sensor in forward and backward directions almost equal. That means the sensor has been aligned parallel with the surface of the workpiece, and the detected shear force at this moment is determined as the sliding frictional force *f*_0_. During the subsequent real-time scratch detection process, when in the uphill state (ii), since *F_y_* >*f*_0_ and *F_z_* >*F*_0_, the robotic arm adaptively moves upward (*Z* > 0) and rotates counterclockwise (*R_x_* > 0) to achieve *F_y_* = *f*_0_ and *F_z_* = *F*_0_. When in the downhill state (iii), since *F_y_* <*f*_0_ and *F_z_* <*F*_0_, the robotic arm adaptively moves downward (*Z* < 0) and rotates clockwise (*R_x_* < 0) to reach the state of *F_y_* = *f*_0_ and *F_z_* = *F*_0_. By this tactile perception–control synergy, the robotic finger can adaptively align with the workpiece surface and make good contact to ensure the precise scratch detection.

Figure 4H presents the sensor responses during the scratch detection process on an uneven car door handle. Firstly, the robotic finger approaches and contacts the handle, and slides along the *y*-axis, which makes the normal force |*F_z_*| and the shear force *F_y_* step up. Simultaneously, the voltage *U_i_* of the interface sensing layer also increases due to the contact with the handle. When the slide just begins, the state between the sensor and the object is in an uphill state (ii) in Fig. 4G, during which *F_y_* >*f*_0_ and *F_z_* >*F*_0_. Therefore, the robotic arm moves upward (*Z* > 0) and rotates counterclockwise (*R_x_* > 0). Subsequently, the robotic finger moves to the downhill state (iii) in Fig. 4G, during which *F_y_* <*f*_0_ and *F_z_* <*F*_0_. Therefore, the robotic arm moves downward (*Z* < 0) and rotates clockwise (*R_x_* < 0). During this slide and scratch detection process, the robotic arm adaptively adjusts its translation (*Z*) in the *z*-axis and its rotation (*R_x_*) along the *x*-axis according to the shear and normal forces, ensuring good contact and parallelly moving along the handle. Simultaneously, the interface sensing layer achieves a precise scratch detection on the handle surface. During the scratch detection process, the surface topography of the workpiece can also be precisely measured by tracking the motion profile of the robot end-effector. This enables the robot to inspect both surface defects and workpiece topography. Repeated full-handle tests (15 scratch encounters) are conducted. All 15 scratches are detected with no misidentification for true scratch and 3 misidentifications for false scratch (detailed in the “Materials and Methods” section). With the multimodal and multidimensional sensory capabilities of the tactile sensor as well as the tactile perception–control synergy, the robot autonomously accomplishes the surface inspection even on irregular workpieces (Movie S2).

### Robotic tactile perception–control synergy for part assembly and socket insertion

Part assembly is a common and challenging task in production lines, where robots need to accurately perceive contact states and apply appropriate forces when performing operations such as screw positioning, insertion, and tightening [45–47]. Currently, industrial robots heavily rely on preset trajectories or fixed force thresholds as criteria in assembly tasks. However, due to the existence of part tolerances, assembly errors, and environmental uncertainties, it often fails to guarantee the reliability and consistency of assembly, especially in fine assembly and flexible production scenarios [48–50].

Here, we combine our tactile sensor with a stereo RGB-D camera (ZED2i) to build a hand–eye coordination of a robotic system consisting of a 6-DoF robot arm (Elite EC66) and a 2-finger gripper (DH-Robotics AG-160-95) for part assembly tasks (Movie S3). Figure 5A illustrates the flowchart of the robotic system in accomplishing a screw-tightening task. First, the robot uses the camera with the Hough circle detection to locate the screws (detailed in Supplementary Notes). Then, leveraging hand–eye calibration (detailed in Supplementary Notes), the robot transforms the screw position from the camera coordinate system to the robotic arm coordinate system, and the robotic claw moves to grasp the screwdriver and pick up the screw. Next, the robot moves to the threaded hole on the part and starts the screwing process. Finally, when the tactile sensor detects that the shear force *F_y_* (or *U_i_*) exceeds the threshold, indicating that the screw is fully tightened, the robot stops screwing.

**Fig. 5.**
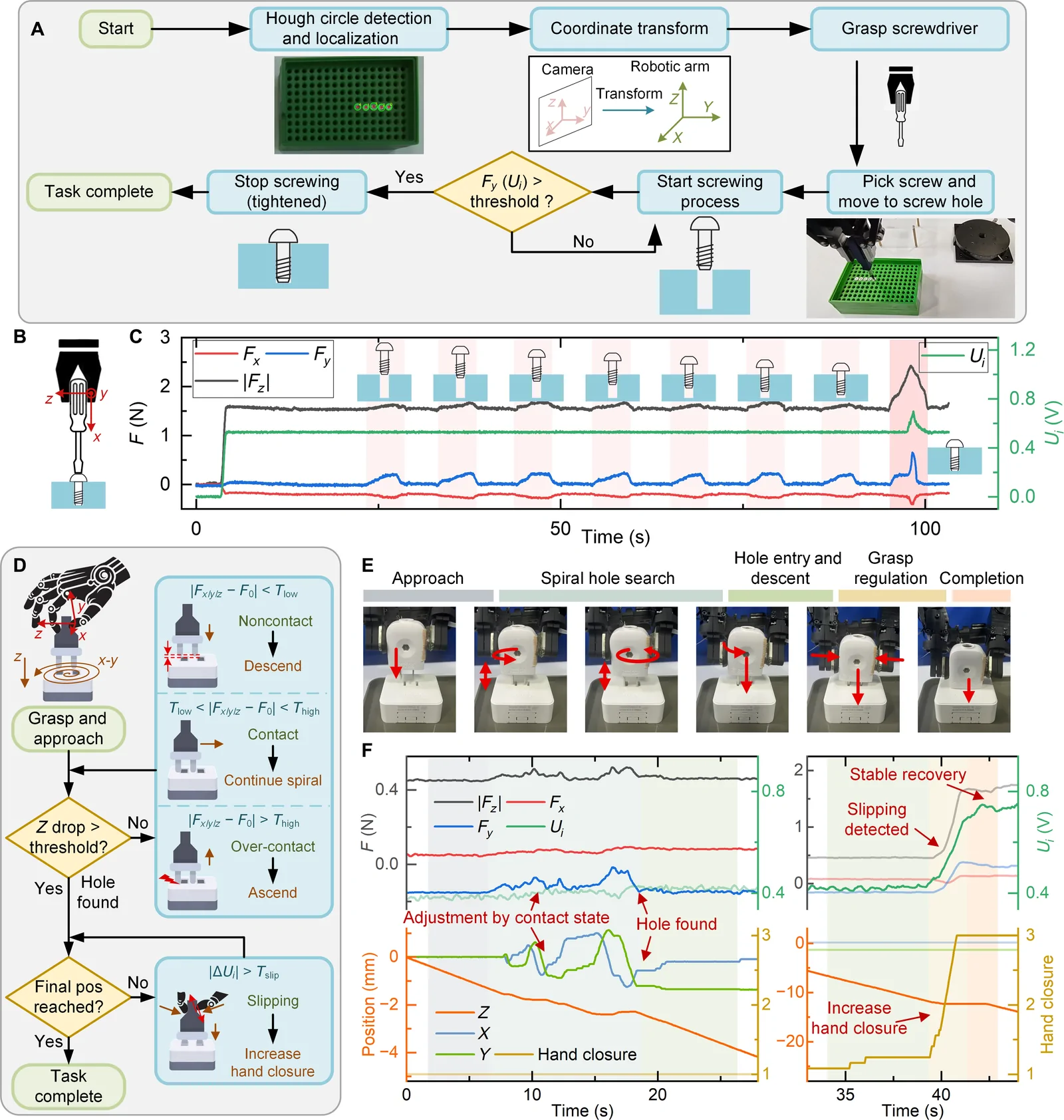
Part assembly and insertion tasks by robotic tactile perception–control synergy incorporated with vision. (A) The flowchart of the robotic system autonomously accomplishing the screw-tightening task. (B) The coordinate system of the tactile sensor equipped on a robotic hand. (C) The interfacial sensing signal and 3-axis force signals detected by the tactile sensor during the screw-tightening task. (D) The flowchart of the robotic system autonomously accomplishing the socket-insertion task. (E) The stages of the socket-insertion task with corresponding snapshots of the robotic operation. (F) The 3-axis force, interfacial sensing, and motion signals of the robotic arm and hand during the socket-insertion task.

Figures 5B and C present the coordinate system, the 3-axis force signals, and the interface sensing signal from the tactile sensor. As shown in Fig. 5C, the pink shadowed area indicates that the robot is performing the screwing action. It is seen that during the screwing process, the shear force *F_x_*, *F_y_*, and normal force *F_z_* increase. This is because *F_x_* provides the vertical frictional force, *F_y_* provides the frictional force in the screwing direction, and *F_z_* provides the supporting force for gripping the screwdriver. Furthermore, since no relative slippage occurs between the robotic hand and the screwdriver, the response *U_i_* of the interface sensing layer remains unchanged during this process. When the screw is fully tightened, the shear forces *F_x_*, *F_y_*, and the normal force |*F_z_*| increase markedly. After tightening, there is relative slippage between the hand and the screwdriver, and the response *U_i_* increases markedly. At this moment, the robot stops tightening and completes the part assembly. With this multimodal and multidimensional tactile sensor and screwing control, the robot enables autonomous accomplishment of part assembly tasks. The tactile-controlled screw-tightening stage is further evaluated in 20 repeated trials, with 19 successful trials (detailed in the “Materials and Methods” section).

We further apply the tactile sensor to a 4-finger dexterous hand (Allegro Hand v4) on a 6-DoF robot arm (Elite EC66) for a socket-insertion task (Movie S4). Figure 5D shows the flowchart of the robotic system. After the vision-guided grasp and approach, the robot switches to a tactile-guided state-conditioned strategy for alignment and insertion (detailed in Supplementary Notes). First, the robot performs a spiral hole search. The controller determines the contact state according to whether the variation of the 3-axis force falls below *T*_low_, lies between *T*_low_ and *T*_high_, or reaches or exceeds *T*_high_, and correspondingly performs *Z* descent, spiral motion in the *X–Y* plane, or *Z* ascent to maintain proper plug–socket contact. Once the plug reaches the hole, the state switches to noncontact, and the robot repeatedly executes the corresponding descent motion until reaching the threshold. After the hole entry, the robot continues downward for insertion. Meanwhile, the interfacial signal *U_i_* is used to detect the slippage between the hand and the plug. Once *U_i_* changes abruptly, the robot increases the hand closure to raise the gripping force, thereby adapting to sockets with different tightness and ensuring successful insertion.

Figures 5E and F present the socket-insertion stages and the corresponding tactile perception–control signals. As shown in Fig. 5F, after the plug contacts the socket surface, the task enters the spiral hole-search stage, during which the shear force *F_y_*, whose direction is closer to the vertical direction in the current configuration, exhibits the most evident variation. When the change in *F_y_* is excessively small or large, the robot mainly adjusts its *Z* position to regulate contact; otherwise, it continues the spiral search motion. Once the hole is found, the 3-axis force signals recover to the values close to their initial levels, indicating the noncontact state and triggering the continuous descent action. During the subsequent insertion, the sudden rise in *U_i_* is accompanied by an increased hand closure command and a marked increase in the normal force *F_z_*, after which the grasp recovers stability as *U_i_* returns to a steady level. The tactile-guided hole-search and insertion stage succeeds in 9 of 10 repeated trials (90%) (detailed in the “Materials and Methods” section). These results demonstrate that the tactile sensor supports both contact-state regulation and adaptive grasp force adjustment for reliable socket insertion.

It is worth mentioning that we have applied the tactile sensor onto 2 distinct robotic platforms (2-finger gripper and 4-finger dexterous hand) to accomplish diverse tasks, further supporting its task-general applicability across robotic deployments.

## Conclusion

We report a flexible fingertip tactile sensor with multimodal and multidimensional perceptions of contact state, matter type, slip/slide, texture/scratch, temperature, and 3-axis force, enabling general-purpose robots accomplishing diverse sophisticated tasks. The sensor comprises an interface sensing layer on the top for multimodal interface sensing and a force sensing layer at the bottom for 3-axis force measurement. The interface sensing layer enables multimodal perceptions of contact state, material type, slip/slide, and texture/scratch, etc. The contact and slip sensing reach ultrafast response (~5 ms), ensuring an instantaneous action trigger of robotic grasp to prevent the object from slipping off. Minute scratches at the ~20-μm scale on various materials, such as aluminum alloy, stainless steel, polytetrafluoroethylene, and resin, are precisely detected by the interface sensing unit, and the correct localization rates range from 90.0% to 100.0% across the 3 scratch-width groups. Furthermore, the integrated 3-axis force sensing layer enables decoupled and high-precision measurements of normal and shear forces, with fast response, low hysteresis, low crosstalk, and excellent repeatability, ensuring fast and dexterous hand manipulation control. The compact tactile sensing architecture and unified thermo-sensation mechanism empower the tactile sensor with the high-integration of multimodality and multidimensionality. A comparison with other representative tactile sensors is shown in Table S2.

Leveraging this multimodal and multidimensional tactile sensor, we further propose a framework of perception–control synergy for robots to accomplish sophisticated tasks, where robots need to precisely perceive and adaptively regulate interactions throughout multistage operations. Such task-level manipulation brings sensory-control complexity: It involves richer multimodal and multidimensional tactile sensing, more diverse interaction states, and long-horizon operations. To address these challenges, we develop a task-level tactile perception–control framework that integrates multimodal and multidimensional tactile perception, task-relevant state interpretation, and adaptive control for robotic sophisticated manipulation. It enables the high-level tactile feedback to regulate interactions throughout the challenging tasks such as scratch/defect inspection, screw assembly, and socket insertion. In the case of scratch inspection, the tactile sensor precisely detects scratches on workpiece surfaces, while the robotic arm adaptively adjusts its translation and rotation according to 3-axis forces detected by the sensor to align the parallel contact. In assembly tasks, such as screw assembly and socket insertion, the robotic system autonomously accomplishes accurate positioning, spiral hole search, insertion, tightening, and 3-axis force control. This tactile perception–control synergy allows reliable, dexterous, and safe operation even in the presence of surface irregularities and environmental uncertainties. These demonstrations cover diverse manipulation scenarios and different robotic platforms, providing the validation that a compact tactile sensing architecture with a perception–control synergy framework can support diverse sophisticated tasks. Further work will focus on its deployment across a broader range of real-world tasks to further expand its generalizability.

## Materials and Methods

### Fabrication of the tactile sensor

Both the interface sensing layer and the pressure sensing layer are fabricated on a flexible polyimide substrate (AP8525R; DuPont Co. Ltd.) by the following steps: (a) Spin coat a 30-μm-thick photoresist (KXN5735-LO; Rdmicro Co. Ltd.) on the polyimide substrate and use lithography to obtain the pattern. (b) Deposit the chromium/platinum (30 nm/120 nm) films sequentially by sputtering and pattern them using a lift-off process. (c) Thermally age the sensors in a vacuum oven (200 °C, 2 h) and then allow to cool naturally to room temperature. (d) Deposit parylene film with a thickness of 6 μm as a protective layer. (e) Electrically age the sensors under 100 °C (aging time 24 h).

The porous material is fabricated by mixing silver nanoparticles (diameter < 100 nm, S110970; Aladdin Co. Ltd.), prepared PDMS solution (Sylgard 184; Dow Corning Company, the ratio of base agent:cross-linker was 10:1 wt.%), and citric acid monohydrate particles (CAM). The volume ratio of silver nanoparticles is 2.5 vol.%. The mass ratio of PDMS:CAM is 1:3.5. After fully stirring the mixture (10 min), the mixture is cured at 75 °C for 3.5 h in a polymethyl methacrylate mold. Then, the mixture is immersed in ethanol for 24 h to dissolve the CAM to form the porous material. Finally, it is washed with deionized water and dried at 70 °C for 1 h.

### Measurement of thermal operating characteristics of the sensor

The power consumption of the active thermal excitation for the sensor is about 50 mW. The start-up time of the sensor is about 30 s (defined as the time when the signal drift within a 10-s window falls below 3*σ* of the signal fluctuation).

The long-term drift of the tactile sensor is evaluated by continuously powering the sensor for 8 h and recording the baseline outputs at 1-h intervals (Fig. S9). The results show the drift errors of 1.8% and 1.1% in force-sensing and interface-sensing outputs, respectively. The influence of ambient temperature on the tactile sensor outputs is shown in Fig. S8.

### Moving window detection method for scratch detection

We use a moving window of 100 samples, selected from the optimization in Fig. S15. We calculate the standard deviation within each window (*σ*_w_) and that of the overall signal (*σ*_a_). A window is identified as containing a scratch when *σ*_w_ >3*σ*_a_; otherwise, it is classified as a non-scratch window. We adopt a conventional 3-sigma (3*σ*) rule commonly used in the sensor field to identify anomalous signal variations. For a normally distributed variable, approximately 99.7% of observations lie within 3 standard deviations of the mean (*μ* ± 3*σ*). We use this 3*σ* criterion to identify the window with pronounced local fluctuations associated with scratches.

The parameter *k* = 3 in 3*σ* rule for scratch detection is further validated by an optimization as shown in Fig. S16. The parameter *k* = 3 achieves the highest TP proportion (correct identification rate) of 95.0%. Lower *k* values increase the false detections, and higher values increase the missed scratches.

### Three-dimensional force calibration

Three-dimensional force calibration is performed using the testing system shown in Fig. S6. The loading direction is defined by an inclination angle *α* and an azimuth angle *β*. Calibration data are collected under normal loading (*α* = 0°) and 16 combined-force loading directions formed by *α* = 10° and 20°, with *β* = 0°, 45°, 90°, … , 315°. For each direction, the applied force is increased stepwise, and the outputs *U*_*p*1_, *U*_*p*2_, and *U*_*p*3_ are recorded. The *F_x_*, *F_y_*, and *F_z_* are obtained by decomposing the force-gauge reading, as illustrated in Fig. S6B and C.

The resulting dataset contains 355 examples, where 60% of the dataset is the training set, 20% is the validation set, and 20% is the test set. The test set is randomly sampled from the 355 dataset and is completely independent from the training set. We use a fully connected neural network for 3-dimensional force calibration. It contains 2 hidden layers, with 5 and 10 neurons in the first and second layers, respectively. The neural network is trained using the Bayesian regularized back-propagation method.

### Configuration of the robot incorporating vision with touch

The robot mainly consists of 2 robotic arms (EC66; ELITE Co. Ltd) mounted on an automated guided vehicle (Oasis-600C; STANDARD Co. Ltd), with one arm equipped with a robotic hand (Allegro Hand; WONIK ROBOTICS) and the other with a 2-finger gripper (AG-160-95; DH-Robotics), together with a binocular depth camera (ZED 2i; Stereolabs Co. Ltd). The signals of the tactile sensors are sampled and transmitted to a PC via a serial communication port and then processed to generate control commands by using the customized compliant control algorithm programmed in Python (Python 3.8.0). Then, the commands are transmitted to the commercial robot controller box via a local area network.

### Repeated robotic task evaluation

For the scratch-inspection task, the robot tests the handle containing 3 scratches in each trial. Five full-handle tests are conducted, corresponding to 15 scratch encounters in total. All 15 scratches are detected, with no misidentification for true scratch and 3 misidentifications for false scratch.

For the screw-assembly task, repeated trials are conducted for the tactile-controlled tightening stage after the screwdriver and screw have been positioned for tightening. A trial is considered successful when the screw is fully tightened and the robot terminates the screwing action according to the tactile feedback. The tightening stage is successfully completed in 19 of 20 trials, corresponding to a success rate of 95%.

For the socket-insertion task, 10 repeated trials are conducted. For each trial, the initial position is randomly selected within a circle of radius 5 mm centered at the nominally aligned position, after which the tactile-guided hole-search and insertion strategy is executed. Nine of the 10 trials result in successful insertion, corresponding to a success rate of 90%.

## Data Availability

The data that support the findings of this study are available from the corresponding author upon reasonable request.
