# Supplementary material for: Multimodal and Multidimensional Tactile Perception for Universal Robotic Sophisticated Tasks
Source: Research (Wash D C). 2026 Nov 6;9:1477. doi: 10.34133/research.1477 (PMC13740327; doi:10.34133/research.1477)
Supplement: Supplementary file 1 [file research.1477.f1.zip › Research_Supplementary Materials_1021_clean.docx]

Supplementary Materials for

**Multimodal and multidimensional tactile perception for universal robotic sophisticated tasks**

Qian Mao^1^†, Yichen Qin^1^†, Zijian Liao^1^, and Rong Zhu^1^*

Corresponding author: zr_gloria@mail.tsinghua.edu.cn

**The PDF file includes:**

Supplementary Notes

Figs. S1 to S16

Tables S1 and S2

**Other Supplementary Materials for this manuscript include the following:**

Movie S1. Summary of Multimodal tactile perception-control synergy for robotic industrial sophisticated tasks.

Movie S2. Perception-control synergy for workpiece defect inspection.

Movie S3. Perception-control synergy for part assembly.

Movie S4. Perception-control synergy for socket insertion.

**Supplementary Notes**

**Three-dimensional force calibration**

To further validate the generalization of the three-axis force sensor to unseen loading directions, we add the held-out direction evaluations. In each evaluation, the samples from the held-out loading direction are utilized as the test sets, and the samples from other loading directions are utilized as the training sets for model calibration. The results are listed in the following Table. The force measurement errors in the held-out evaluations are slightly larger than the result of the random sample-wise split because the calibrated model lacks the whole coverage of certain representative directions.

In addition, we validate the generalization of the three-axis force calibration in the demonstrations of the robotic tasks, including scratch inspection, screw assembly, and plug-socket insertion. During these robotic tasks, the contact forces vary along various loading directions and force trajectories independent from the calibration datasets. The sensor can accurately respond to the interactions and support the tactile feedback controls, which further demonstrate the sensor generalization to the unseen combined-force conditions.

**Table. Three-axis force measurement in held-out direction evaluations**

| Test set | RMSE of Fz (N) | RMSE of Fx (N) | RMSE of Fy (N) |
| --- | --- | --- | --- |
| Held-out direction 1  (α = 20° and β = 315°) | 0.45 | 0.27 | 0.23 |
| Held-out direction 2  (α = 10° and β = 270°) | 0.21 | 0.20 | 0.29 |
| Held-out direction 3  (α = 10° and β = 315°) | 0.13 | 0.46 | 0.18 |
| Random  sample-wise split | 0.16 | 0.17 | 0.18 |

Currently, each fabricated sensor needs to be calibrated individually.

**The control law for scratch detection**

To achieve good contact between the sensor and the uneven surface, the movement of the robotic arm needs to be controlled using the three-dimensional force measured by the sensor. Assuming that the relationship between the tactile sensor and the workpiece follows a virtual spring-damping kinetic mechanism:

$$\begin{aligned} \varphi_{total}=\varphi-\varphi_{des}-\alpha\cdot\Delta\eta-\beta\cdot\dot{\eta} \end{aligned}$$

where $\varphi_{total}$ is the total force, $\varphi$ is the normal or shear force between the tactile sensor and the workpiece, $\varphi_{des}$ is the desired normal or shear force between the tactile sensor and the workpiece. $\Delta\eta$ is the translational distance or rotational angle of the X/Y/Z direction. $\dot{\eta}$ is the velocity or angular velocity of the X/Y/Z direction. $\alpha$and $\beta$ are the stiffness coefficient and damping coefficient of the spring-damping kinetic system.

When the sensor maintains stable and good contact with the workpiece, the total force $\varphi_{total}$≈0. Therefore, the translational distance or rotational angle of the X/Y/Z direction can be calculated by:

$$\begin{aligned} \Delta\eta=\frac{1}{\alpha}\left( \varphi-\varphi_{des} \right)-\frac{\beta}{\alpha}\cdot\dot{\eta} \end{aligned}$$

Based on the above derivation, the following control law can be obtained:

$$\begin{aligned} Z=k_{1}\left( F_{z}-F_{0} \right)+\mu_{1}\dot{Z} \end{aligned}$$

$$\begin{aligned} R_{x}=k_{2}\left( F_{y}-f_{0} \right)+\mu_{2}\dot{R_{x}} \end{aligned}$$

$$\begin{aligned} R_{y}=k_{3}\left( F_{x}-f_{0} \right)+\mu_{3}\dot{R_{y}} \end{aligned}$$

where $Z$, $R_{x}$, and $R_{y}$ are translation in the z-direction and rotation in the x-axis and y-axis, respectively. *k*_1_, *k*_2_, *k*_3_, *μ*_1_, *μ*_2_, and *μ*_3_ are coefficients related to the robot arm control. $\dot{Z}$, $\dot{R_{x}}$, and $\dot{R_{y}}$ are the velocity in the Z direction, and the rotational angular velocities in the X and Y directions, respectively. *F*_0_ and *f*_0_ are the support force and the sliding friction force when the sensor and the object are in good contact, respectively.

In this car door handle case, *k*_1_, *k*_2_, *k*_3_, *μ*_1_, *μ*_2_, *μ*_3_, *F*_0,_ and *f*_0_ are set as 0.1, 0.00035, 0.00035, 0, 0, 0, 1, and 1.5, respectively.

**Hand-eye calibration**

To localize detected screws in the robot base frame, we calibrate the rigid transformation between the ZED 2i and the robot end-effector. The ZED 2i camera is mounted on the robot wrist in an eye-in-hand configuration. We use a planar chessboard calibration target with 9 × 6 inner corners (square size 25 mm) fixed in the workspace. We collect 76 pose pairs by moving the robot to different viewpoints while observing the static chessboard. For each viewpoint *i*, we record the end-effector pose in the base frame$\mathbf{H}_{ee,i}^{base}$from robot kinematics, and estimate the target pose in the camera frame$\mathbf{H}_{cal,i}^{cam}$by detecting the chessboard corners and solving a PnP problem using the known 3D corner geometry and the camera intrinsics. Hand-eye calibration is then formulated as the standard$\mathbf{AX}=\mathbf{XB}$ problem:

$$\mathbf{A}_{ij} \mathbf{H}_{cam}^{ee}=\mathbf{H}_{cam}^{ee} \mathbf{B}_{ij} \mathbf{A}_{ij}=\left( \mathbf{H}_{ee,j}^{base} \right)^{-1} \mathbf{H}_{ee,i}^{base} \mathbf{B}_{ij}=\mathbf{H}_{cal,j}^{cam} \left( \mathbf{H}_{cal,i}^{cam} \right)^{-1}$$

where$\mathbf{H}_{cam}^{ee}$is the unknown camera-to-end-effector transform. We solve for$\mathbf{H}_{cam}^{ee}$using OpenCV’s hand-eye calibration implementation, with the Daniilidis solver option.

**Screw position detection**

We detect screw-head positions in the ZED 2i RGB images using a Hough circle-based pipeline. For each incoming frame from the ZED 2i, we first convert it to grayscale and apply contrast enhancement via CLAHE (clipLimit = 2.0, tileGridSize = 8 × 8), followed by Gaussian smoothing (kernel size = 3 × 3) to reduce sensor noise and local texture. Circular candidates corresponding to screw heads are then extracted using the gradient-based Hough transform implementation in OpenCV. In post-processing, we suppress false positives caused by the holes from the screw tray using a center-intensity validation: we compute the mean grayscale intensity within a concentric disk of radius 0.25*r*, and only retain candidates whose mean intensity exceeds a fixed threshold of 80.

Next, based on the detected screw center (*u, v*), we combine the corresponding depth *z* and the camera intrinsic matrix **K** to compute its 3D position in the camera frame via back-projection:

$$\mathbf{p}_{cam}=z \mathbf{K}^{-1}\left[ \begin{matrix} u \\ v \\ 1 \end{matrix} \right]$$

where the intrinsic matrix **K** is provided by the ZED 2i built-in self-calibration method. Finally, we map the 3D screw position from the camera frame to the robot base frame by chaining the current end-effector pose$\mathbf{H}_{ee}^{base}$and the calibrated hand-eye transform$\mathbf{H}_{cam}^{ee}$.

$${\tilde{\mathbf{p}}}_{base}=\mathbf{H}_{ee}^{base} \mathbf{H}_{cam}^{ee} {\tilde{\mathbf{p}}}_{cam} \tilde{\mathbf{p}}=\left[ x,y,z,1 \right]^{T}$$

**Vision-based grasp and approach for socket insertion**

For the socket-insertion task, the plug and socket are first localized from the RGB-D input. We provide the mesh models of both objects in advance. Then the robot uses SAM2 and FoundationPose to estimate their 6-DoF poses. The target pose of the robot end-effector for either plug grasping or socket approach is computed as:

$$\mathbf{H}_{ee}^{base}=\mathbf{H}_{cam}^{base}\mathbf{H}_{obj}^{cam}\mathbf{H}_{ee}^{obj}$$

where $\mathbf{H}_{obj}^{cam}$ is the estimated object pose in the camera frame, $\mathbf{H}_{cam}^{base}$ is the calibrated camera-to-base transformation, and $\mathbf{H}_{ee}^{obj}$ is the predefined relative pose of the end-effector with respect to the object. The hand grasp pattern and the transfer trajectory are predefined. The corresponding arm motion is then solved to execute plug grasping and coarse approach to the socket.

**Force-guided spiral hole-search policy for socket insertion**

After vision-based grasp and approach, the robot enters a force-guided surface hole-search stage to localize the socket hole. At each control cycle *k*, the controller determines the surface contact state from the three-dimensional force vector $\mathbf{F}_{k}=\left[ F_{x,k},F_{y,k},F_{z,k} \right]^{\top}$, updates the target end-effector pose accordingly, and terminates the search once the hole-entry criterion is satisfied. The controller is executed at a fixed frequency $f=20 Hz$.

The first $N_{b}$ valid force values are averaged to obtain the baseline force $\bar{\mathbf{F}}$. The force variation is defined as:

$$\Delta\mathbf{F}_{k}\boldsymbol{=}\mathbf{F}_{k}\boldsymbol{-}\bar{\mathbf{F}}$$

The surface contact state is then determined from the maximum absolute force variation:

$$\left\| \Delta\mathbf{F}_{k} \right\|_{\infty}=\max\left( \left| \Delta F_{x,k} \right|,\left| \Delta F_{y,k} \right|,\left| \Delta F_{z,k} \right| \right)$$

$$s_{k}=\left\{ \begin{matrix} \text{non-contact}\text{ }, & \left\| \Delta\mathbf{F}_{k} \right\|_{\infty}<T_{\text{low}} \\ \text{contact}\text{ , } & {T_{\text{low}}\leq\left\| \Delta\mathbf{F}_{k} \right\|}_{\infty}\boldsymbol{<}T_{\text{high}} \\ \text{over-contac}\text{t , } & \left\| \Delta\mathbf{F}_{k} \right\|_{\infty}{\geq T}_{\text{high}} \end{matrix} \right.$$

Under the contact state, the controller advances the search in the X-Y plane, while the Z direction is used for normal contact regulation. The end-effector orientation is fixed during the search. The planar search trajectory is an Archimedean spiral:

$$r\left( \theta\right)=r_{0}+b\left( \theta-\theta_{0} \right) x\left( \theta\right)=r\left( \theta\right)\cos\theta y\left( \theta\right)=r\left( \theta\right)\sin\theta b=\frac{p}{2\pi}$$

To achieve approximately uniform spatial sampling, the spiral is discretized by arc length:

$$\Delta s=\frac{v_{s}}{f}$$

Let $\mathbf{p}_{i}^{\text{sp}}=\left[ x_{i},y_{i},0 \right]^{\top}$ denote the $i$-th spiral offset and let $z_{k}$ denote the accumulated Z offset relative to the initial search pose. The commanded target position $\mathbf{p}_{k+1}^{*}$ is updated according to the current contact state $s_{k}$:

$$\mathbf{p}_{k+1}^{*}=\left\{ \begin{matrix} \mathbf{p}_{0}+\mathbf{p}_{i_{k}}^{sp}+\left[ 0,0,z_{k}-\delta_{z} \right]^{\top}, & s_{k}=\text{non-contact} \\ \mathbf{p}_{0}+\mathbf{p}_{i_{k}+1}^{sp}+\left[ 0,0,z_{k} \right]^{\top}, & s_{k}=\text{contact} \\ \mathbf{p}_{0}+\mathbf{p}_{i_{k}}^{sp}+\left[ 0,0,z_{k}+\delta_{z} \right]^{\top}, & s_{k}=\text{over-contact} \end{matrix} \right.$$

where $\mathbf{p}_{0}$ is the initial search position, $i_{k}$ is the current spiral index, and $\delta_{z}$ is the normal adjustment step. Thus, lateral exploration is executed only in the contact state, whereas non-contact and over-contact trigger downward and upward normal corrections, respectively.

Hole entry is determined by the cumulative downward displacement after the first contact. Let $z_{\text{fc}}$ denote the Z-axis position at the first instant satisfying $s_{k}\in\{\text{contact}, \text{over-contact}\}$. Hole entry is declared when:

$$z_{\text{fc}}-z_{k}\geq T_{drop}$$

In our implementation, the policy parameters are set to $N_{b}=50$, $T_{\text{low}}=0.04 N$, $T_{\text{high}}=0.12 N$, $p=0.5 \mathrm{mm}$, $v_{s}=2 \mathrm{mm}/s$, $\delta_{z}=0.01 \mathrm{mm}$ and $T_{drop}=3 \mathrm{mm}$.

**Grasp adjustment policy for socket insertion**

After hole entry, the controller switches to a slip-triggered insertion stage, in which abrupt changes in the interfacial signal $U_{i}$ are used to detect relative slip between the hand and the plug. The same control frequency *f* as defined above is used throughout this stage. Let $U_{i}\left( k \right)$ denote the sampled interfacial signal at control cycle k. A moving average over the previous *M* samples is computed as:

$$\bar{U}_{i}\left( k \right)=\frac{1}{M}\sum_{j=k-M}^{k-1} U_{i}\left( j \right)$$

and the slip state $s_{k}$ is determined by:

$$s_{k}=\left\{ \begin{matrix} \mathrm{slip}, & \left| U_{i}\left( k \right)-\bar{U}_{i}\left( k \right) \right|>T_{s} \\ \text{non-slip}, & \text{otherwise}\text{ } \end{matrix} \right.$$

To regulate the grasp, we define a scalar hand-closure command $c_{k}\in\left[ c_{min},c_{max} \right]$, where $c_{min}$ corresponds to the initial grasp posture and $c_{max}$ corresponds to the maximum grasp posture. The grasp posture is predefined as 16-dimensional joint targets of the dexterous hand, denoted by $\mathbf{q}^{init}\in\mathbb{R}^{16}$ and $\mathbf{q}^{max}\in\mathbb{R}^{16}$, respectively. The commanded hand joint target $\mathbf{q}_{h}\left( k \right)$ is obtained by linear interpolation:

$$\lambda_{k}=\frac{c_{k}-c_{min}}{c_{max}-c_{min}} \mathbf{q}_{h}\left( k \right)=\left( 1-\lambda_{k} \right)\mathbf{q}^{init}+\lambda_{k}\mathbf{q}^{max}$$

When slip is detected, the arm insertion motion is temporarily paused and the hand-closure command is increased by a fixed increment $\delta c$. The corresponding update law is:

$$c_{k+1}=\left\{ \begin{matrix} \min\left( c_{max},c_{k}+\delta c \right), & s_{k}=slip \\ c_{k}, & s_{k}=\text{non-slip} \end{matrix} \right.$$

$$\mathbf{p}_{k+1}=\left\{ \begin{matrix} \mathbf{p}_{k}\boldsymbol{,} & s_{k}=\mathrm{slip} \\ \mathbf{p}_{k}-\delta_{ins}\mathbf{n}_{ins}, & s_{k}=\text{non-slip} \end{matrix} \right.$$

where $\mathbf{p}_{k}$ is the target end-effector position, $\mathbf{n}_{ins}$ is the unit insertion direction (Z), and $\delta_{ins}=v_{z}/f$ is the insertion step determined by the commanded insertion speed $v_{z}$ and the control frequency *f*.

In our implementation, $M=20$, $T_{s}=0.1 \mathrm{mV}$, $c_{min}=1$, $c_{max}=3$, $v_{z}=1 \mathrm{mm}/s$, and $\delta c=0.08$.

**Figure S1. Circuit routing of the FPCB.** (a) Wire and the sensor arrangement of the interface sensing layer on the FPCB. (b) Wire and the sensor arrangement of the pressure sensing layer on the FPCB.

**Figure S2. The schematic diagram of the conditioning circuit.**

**Figure S3. The schematic diagram of the fabrication process.** (a) Fabrication processes of the interface sensing layer and the pressure sensing layer. (b) Fabrication process of the porous material.

**Figure S4. Photograph of the tactile sensor and its components.**

**Figure S5. Detection limits of three-axis force.** (a) The detection limit of *F_z_*. (b) The detection limit of *F_x_*, while *|F_z_*| = 0.1 N is applied. (c) The detection limit of *F_y_*, while *|F_z_*| = 0.1 N is applied. The detection limits of *F_z_*, *F_x_*, *F_y_* are 0.003 N, 0.017 N, and 0.018 N, respectively. The detection limit is tested by putting a small weight (e.g., a screw) onto the sensor. The minimum applied force whose sensing response exceeds three standard deviations (3σ) of the baseline signal of the sensor is taken as the detection limit.

**Figure S6. The testing system for the three-axis force sensing.** (a) The photograph of the testing device. (b)-(c) The schematic diagram of the three-dimensional force calculation.

**Figure S7. Crosstalk among the three force components.** “err F_x_” refers to the error of *F_x_*, and the others are similar. The results show the error of the sensor when combined loads are applied. For example, the graph in the first row and third column shows the measurement error of the force *F_z_* when the applied force *F_x_* varies from -5 to 5 N. The graph in the third row and the first column shows the measurement error of the force *F_x_* when the applied force *F_z_* varies from -20 to 0 N.

**Figure S8. Sensors’ responses under different temperatures varying from 25 ℃ to 45 ℃.** Error bars are standard deviations of three repeated measurements of one specific sensor.

**Figure S9. Long-term drift of the interface sensing output U_i_ and the force sensing outputs U_p1_, U_p2_, U_p3_ over 8 hours.**

**Figure S10.** **Influence of contacted material on the force-sensing responses.** (a)-(c) Responses of *U_p1_*, *U_p2_*, and *U_p3_* when contacting with different materials (PE and PPS).

**Figure S11. Influence of normal contact force on the interface-sensing response.** Interface signal *U_i_* as a function of normal contact force when contacting PE and PPS. Points represent the means of three repeated measurements, and error bars indicate standard deviations.

**Figure S12. Photographs of scratches.** (a) Photographs of scratches on the aluminum alloy. (b) Photographs of scratches on the stainless steel. (c) Photographs of scratches on the teflon. (d) Photographs of scratches on the resin. We create the scratches by manually dragging the blades with different tip size on the sample surfaces. The resulting samples retain the irregular geometries and edge features generated by scratching, making them similar as the real surface damages encountered in practice.

**Figure S13. Interface responses to the same scratch (width: 74.2 μm) on aluminum alloy at the beginning (a) and the end (b) of repetitive scratch detections (90 trials in total).**

**Figure S14. The schematic diagram of the control system for workpiece defect inspection.**

**Figure S15. The optimization of the window length.**

**Figure S16. The optimization of the parameter *k* in three-sigma (3σ) rule for scratch detection.** TP, FP, and FN denote correct scratch identifications, false scratch identifications, and missed scratches, respectively. k = 3.0 and 3.5 yield the highest correct identification rate of 95.0%.

**Table S1. Thermal properties of some materials**

| Material | Thermal conductivity (W/(m·K)) |
| --- | --- |
| PU | 0.180 |
| PPS | 0.210 |
| PEEK | 0.250 |
| PA | 0.290 |
| PE | 0.331 |
| Bakelite | 0.377 |
| Steel | 16.3 |
| Brass | 109 |

**Table S2. Comparison with other representative multimodal and multidimensional tactile sensors**

| **Work** | **Principle** | **Tactile modalities** | **Response speed** | **Crosstalk metric** | **Robotic use** |
| --- | --- | --- | --- | --- | --- |
| **[6], 2024** | Magnetoelastic | Normal/shear, tap/vibration, location, shape/texture | N.R. | N.R. | Underwater recognition |
| **[35], 2024** | Capacitive + triboelectric + piezoresistive | 3-axis force, contact/slip, proximity, material/hardness/texture | TENG: 38 ms; pressure: 85 ms | N.R. | Recognition; manipulation-state sensing |
| **[22],**  **2025** | Triboelectric | Pressure, temperature | Pressure: 70 ms; temperature: 67 ms | P–T crosstalk <0.4% / 3.2% | Recognition; teleoperation |
| **[5], 2026** | Visuotactile + triboelectric + inertial | force, temperature, proximity, texture/material, slip/vibration | N.R. | N.R. | HRI; sorting; tactile search |
| **[36], 2026** | FBG + visuotactile | 3-axis force, contact geometry, hardness, slip | N.R. | N.R. | Classification; delicate manipulation |
| **Our work** | **Thermo-sensation** | **3-axis force, contact, material, slip/slide, texture/scratch, temperature** | **Contact: 6 ms; slip: 5 ms; force: 44 ms** | **Force-axis <2.3%; inter-layer <4.0%** | **Task-level sophisticated manipulation: inspection; tightening; insertion** |

N.R., not reported.

Movie S1.

Summary of Multimodal tactile perception-control synergy for robotic industrial sophisticated tasks.

Movie S2.

Perception-control synergy for workpiece defect inspection.

Movie S3.

Perception-control synergy for part assembly.

Movie S4.

Perception-control synergy for socket insertion.
